# Joint Durability of Steam-Treated Beech Wood

**DOI:** 10.3390/polym15153318

**Published:** 2023-08-06

**Authors:** Goran Mihulja, Dominik Poljak, Tomislav Sedlar

**Affiliations:** 1 Faculty of Forestry and Wood Technology, University of Zagreb, 10000 Zagreb, Croatia; gmihulja@sumfak.unizg.hr; 2 Drvodjelac d.o.o., 42240 Ivanec, Croatia; poljak@drvodjelac.hr

**Keywords:** steamed beech wood, ecological modification, wood stability, bonding strength, bonded joint durability

## Abstract

Steaming beech wood is one of the most commonly used eco-based processes for wood color equilibration. In addition to color equalization, steaming has also been noticed to stabilize the final product (solid wood panels). The beech wood samples used in this study were steamed for two different periods. PVAc and polyurethane (PU) adhesives were used to analyze bonding strength and durability. The bonding strength was measured according to the EN 13354 standard. The samples were treated before testing according to the first part of the standard, i.e., immersion in water. The durability of the bonded joint was tested according to the ISO 9142 standard. The samples were treated before testing with two methods. The results of the bonding strength show the influence of the steaming process on the bonded joint. Short exposure to steam decreased bonding strength, and prolonged exposure increased bonding strength. From the results given and the statistical analysis, it can be concluded that a prolonged steaming period increases the stability of the beech wood and thus the durability of the bonded joints.

## 1. Introduction

The gluing of wood occupies an important part of the technological process [1,2,3]. As wood prices are constantly rising as raw materials and their quality is decreasing, the interest in solid wood products is growing, and the desire to properly use wood raw materials is increasing, leading to the introduction of various wood adhesive processes. The complexity of woodcraft and the bonding process into a whole composite open up a wide area of research.

Steaming wood can be the first step in this complex and demanding task. Steaming wood began to be used in practice to even out the color, primarily in wood with an incorrect heartwood. Wood steaming is a process in which the cold wood is exposed to saturated water vapor, as a result of which the wood heats up and its properties change. This is the process that can ensure positive effects such as uniformization of color, reduction of wood deformations, increase in wood stability in the drying process, and reduction of errors on glued panels [4,5,6,7,8,9]. Color changes are related to specific chemical properties of different intensities and complexity, which can affect the main chemical components and secondary components of wood in relation to the actual treatment conditions (temperature, time, and treatment environment) [10,11,12,13]. The steaming process partly reduces the internal growth stresses of the wood, which decreases the potential for cracking and deformations during the drying process [4]. In addition to these beneficial effects, steaming also prevents biological attacks, promoting wood sterilization before it enters a drying oven [5,14].

In the production of solid wood panels, the problem of the appropriate preparation of the wood is to produce high-quality products with as little loss of firmness as possible [15]. In the context of wood gluing, the problem of numerous influential factors that act on wood is expressed, changing its ability to interact with adhesives and adhesive systems. Auspicious effects can be achieved by hydrothermal treatment of green wood [16]. However, there can also be unwanted side effects [17]. Loss of strength is one of the main disadvantages of thermal treatment. The chemical structure [18] and changes in wood components play an important role in the wood’s strength when exposed to high temperatures [19]. The reduction in wood resistance seems to be linked to the removal of secondary chain hemicellulose constituents [20,21].

In the wood gluing framework, numerous factors affect wood and its ability to interact with adhesives and adhesive systems. Due to variability in wood sources, the desire for better performance, and cost-saving tendencies, the manufacturing of bonded wood products is constantly challenged [22,23]. To achieve strong and durable bonds, the resin must properly saturate the wood surface and cure it efficiently to ensure sufficient strength and deformation [24]. The moisture content of the wood, pH, buffering capacity, and extractives affect the wetting of adhesive on the surface [25].

The beech wood (*Fagus sylvatica* L.) used in this study is the most common hardwood in Europe and the standard wood species quoted in European standards. Due to its good mechanical and technical properties, it is widely used in all branches of the wood processing industry (the production of veneer, furniture, massive wood panels, etc.) [26,27,28]. Beech wood is a diffusely porous wood with fine and numerous burrs, which evenly potentiate the distribution of stress between samples of the material [29].

Wood is a hygroscopic material; i.e., with a change in the humidity and temperature of its surroundings, it shrinks and swells. As a result of changes in dimensions, stress forms in joints when any kind of adhesive is used. For this reason, when selecting adhesives, the physical and mechanical properties of the adhesive must be as close as possible to those of the wood. Therefore, the joints are under less stress from the characteristic dimensional change of the wood. Considering all that and the potential of this research’s results on its usage in the industry, it leads to adhesive choice. The best is to use PVAC due to its wide usage for dry and semi-dry conditions. For semi-dry to wet conditions, it can be used more durometric adhesives, but to be on the future side of production, self-apposing is PUR adhesive, which is used more and more every day.

The property of a joint to retain its strength over time is the fundamental feature of the present investigation. The focus is on the dependence of the bonding process, the properties of the adhesives used, and the properties of the wood. Therefore, the purpose and goals of this research are as follows:

- Determine the strength of the joints of modified beech wood depending on the different steaming processes;
- Investigate and define different adhesives to achieve sufficient joint strength in modified beech wood;
- Define the types of joint durability of modified beech wood related to different adhesives;
- Determine the optimal steaming process for beech wood.

## 2. Materials and Methods

For this research, log samples of 3 meters in length, class quality F, I, and II, were selected from the Bjelovar-Bilogora County habitat in Bilogora, Croatia. The logs were first sawn into 50 mm thick planks and then into samples with dimensions of 32 × 50 × 250 mm. Half of the raw samples were then exposed to direct steaming in an industrial steamer at a constant steam temperature of 99 °C and a pressure of 0.15 MPa. A control sample was not steamed but was placed in a dryer together with the steamed samples after pre-drying. No deficiencies or wood defects were detected in the samples produced and processed this way.

In this research, well-known and tested adhesives for finger joints and buttock joints were used: Multibond EZ-1 PVAc adhesive and Klebit PUR 501.

Based on experience, the results of tests on joint strength and durability have a relatively high standard deviation. Due to the large number of test samples and types of experiments, a particular marking system was developed for more convenient monitoring during the tests and continuous adoption of conclusions, as shown in Table 1.

### 2.1. Method to Measure the Strength of the Bonded Joint

Test pieces were produced to measure the strength of a bonded joint according to EN 13354 [30]. Beech wood samples (32 × 50 × 270 mm) were conditioned at a temperature of 23 ± 2 °C and a relative humidity of 50 ± 5% (from now on “laboratory conditions”) for seven days. Before bonding, the glue surfaces were processed using a planer, and the wood panel was bonded from five samples to a dimension of 32 × 250 × 270 mm. The bonding process was carried out with an adhesive application of 200 g/m^2^ (the adhesive manufacturer prescribed 175–250 g/m^2^ for Multibond EZ-1 and 100–200 g/m^2^ for PUR 501), under laboratory conditions, after which the panels were conditioned again for seven days.

Ten wood panels were assembled for control samples and ten for each steaming regime. Before sawing the test pieces, the panels were planed to a thickness of 25 mm. After planing, six test pieces were sawn from each plate according to the EN 13354 standard [31]. When the test pieces were prepared, special attention was paid to positioning the bond line precisely in the middle of the probe. Plates were sawn into strips parallel to the bond line, and then the test pieces were sawn from those strips according to the prescribed standard. Finally, notches were made using a 3 mm wide flat-tooth circular saw blade.

Additional reliability of the results was achieved by measuring the dimensions of all prepared test pieces with the aim of more accurate calculations of the bonding strengths.

Half of all test pieces (control test pieces and both steaming regimes) were tested according to the ISO 6238 standard [31] on a universal testing machine, the Shimadzu AG-X 100 (Shimadzu Corporation, 604-8442 Kyoto, Nakagyo Ward, Nishinokyo Kuwabaracho, 1, Japan) (from now on UTM) after conditioning. The other half of the test pieces were sorted according to the recommendation of EN 13353 [32] (according to their purpose, that is, by type of adhesive):

- Samples bonded with Multibond EZ-1 adhesive (PVAc adhesive) intended for dry conditions, i.e., samples immersed in water at 20 °C for 24 h prior to testing;
- Samples bonded with PUR 501 adhesive (PUR adhesive) were intended for humid conditions, that is, the samples were cooked in distilled water for 6 h and then immersed in water at 20 °C for 1 h before testing.

It has been established that adhesive-bonded samples for humid and outside conditions cannot be treated according to the recommendation of this standard (number of total test pieces designation); all samples were treated solely according to the first point of this standard, that is, they were submerged in water at 20 °C for 24 h prior to testing.

### 2.2. Testing the Durability of the Bonded Joint According to ISO 9142 Standard [33]—Cycle D6

The bonded joint was carried out on test pieces made according to the ISO 6238 standard [31]. As suggested by the ISO 9142 standard [33], cycle D6 was used to test the durability of the bonded joint. Cycle D6 consists of two phases:

(a) Impregnation of samples by distilled water using vacuum pressure (Laboratory Impregnation Chamber, Kambič, Metliška cesta 16, 8333 Semič, Slovenia—EU) at a temperature of 23 ± 2 °C;
(b) Exposure of the samples to climate conditions, that is, a relative air humidity of 30 ± 5% and a temperature of 23 ± 2 °C, for ten days.

Phase a. consists of the following steps:

1. Submersion of the samples in distilled water and vacuum under a pressure of 0.092 MPa for 15 min;
2. Change in pressure in the cylinder, that is, samples were placed under a pressure of 0.6 MPa for two hours;
3. Change in pressure to 0.092 MPa for 15 min;
4. Change in pressure to 0.6 MPa for two hours.

**Figure 1:**
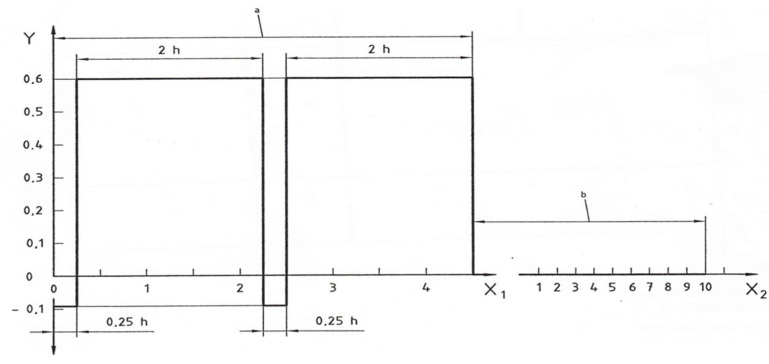
Graphical representation of the sample treatment according to the ISO 9142 standard [33]—Cycle D6. (X_1_—exposure period (h); X_2_—exposure period (days); Y—over/under pressure (MPa); a—exposure period in chamber A: 4 h + 30 min.; b—exposure period in chamber B: 10 days at (30 ± 5) % R.H.).

Phase b. is the continuation of the treatment; that is, after being placed at a pressure of 0.6 MPa, the samples were exposed to a relative air humidity of 30 ± 5% and a temperature of 23 ± 2 °C under ambient air pressure. A graphical representation of the flow of sample impregnation is shown in Figure 1.

### 2.3. Testing the Durability of the Bonded Joint Using the Simulation of Environmental Conditions Method

The standard EN 13353 [32] method of accelerated aging of bonded joints by treatment with water and boiling was inappropriate to observe the modifications due to the complete collapse of all bonded joints. For this reason, a new, highly intensive, and applicable method of accelerated aging of bonded joints was developed for this research. The test samples were exposed in a plastic chamber with artificial air circulation to a relative air humidity of 70 ± 5% at a temperature of 23 ± 2 °C controlled by a mixture of salt and water (NaCl + H_2_O).

**Figure 2:**
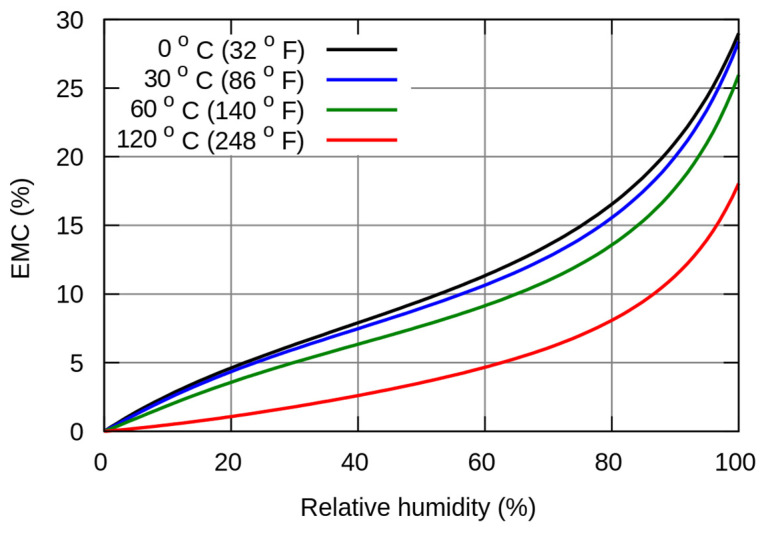
The Hailwood–Horrobin equilibrium moisture content (EMC) graph [34].

The mass of the samples was repeatedly measured according to the ISO 6238 standard [31] to determine the time required for the samples to achieve equilibrium moisture content (EMC). According to the Hailwood–Horrobin diagram (Figure 2) [34], EMC should, under these conditions, be ~13% of the water content in the test samples. Therefore, very intense wood deformation was caused (in the shortest time possible) through natural moisture absorption and, consequently, the stress in the bonded joint, without the negative effects of free water activity that never occur in the normal life cycle of the product.

Subsequently, the samples were subjected to intensive drying processes in a plastic chamber with inside artificial air circulation at a relative air humidity of 30% at a temperature of 23 ± 2 °C, controlled by magnesium chloride hexahydrate (MgCl_2_ + 6H_2_O). Before bonding strength testing on a UTM, the test pieces were subjected to a second cycle under the same wetting and drying conditions.

### 2.4. Statistical Analysis

Statistical analysis and data visualization were performed using the Statistica (ver. 7.1) software. An analysis of variance (ANOVA) was used to evaluate the variations tested. The sources of variation are presented in Table 1. The Shapiro–Wilk test confirmed the normality of the data, and Levene’s test checked the homogeneity of the variances. When the assumption of homogeneity was met, a one-way ANOVA test was used; if not, the Kruskal-Wallis test [35,36] was used. 

An appropriate pairwise comparison Scheffe (post hoc) test [37] was performed if the Kruskal–Wallis test proved statistical significance. Thus, the Student’s pairwise test (equal variances) was used after the one-way ANOVA, while the Games-Howell pairwise test (non-equal variances) was used after Welch’s ANOVA. The effect sizes of the pairwise test were estimated using holm-adjusted *p*-values. Graphic displays were made using box whiskers and bar plots. Descriptive statistics were also used to display the results on graphs. Boxes show the 25th and 75th percentiles (interquartile—IQR), the whisker lines show the minimum and maximum values (±1.5*IQR), square points denote the arithmetic mean, and post hoc tests are displayed in tables showing significant differences between pairs with bold letters of p values at the 5% significance level. All results are also shown with a 95% family-wise confidence level.

## 3. Results and Discussion

### 3.1. Bonding Strength

The bonding strength was measured using two methods. Analysis of the results obtained can be used to determine and/or detect trends in the modification effect of the samples on the strength of the PVAc adhesive joints. 

**Figure 3:**
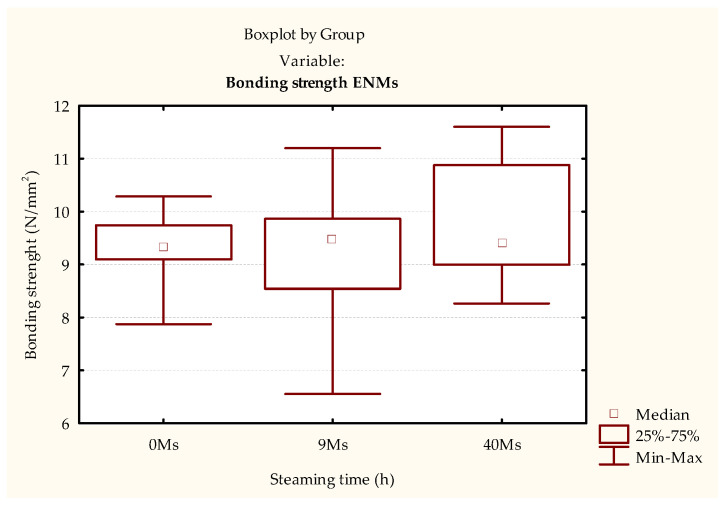
Results of testing conditioned test pieces bonded with PVAc adhesive.

The initial bond strength of the joints bonded with PVAc adhesive gives an idea of the condition achieved at the beginning of their life cycle. Since there are no statistically significant differences, the result that can be pointed out is that steem modification does not change the bond strength in dry conditions (Figure 3 and Table 2).

**Figure 4:**
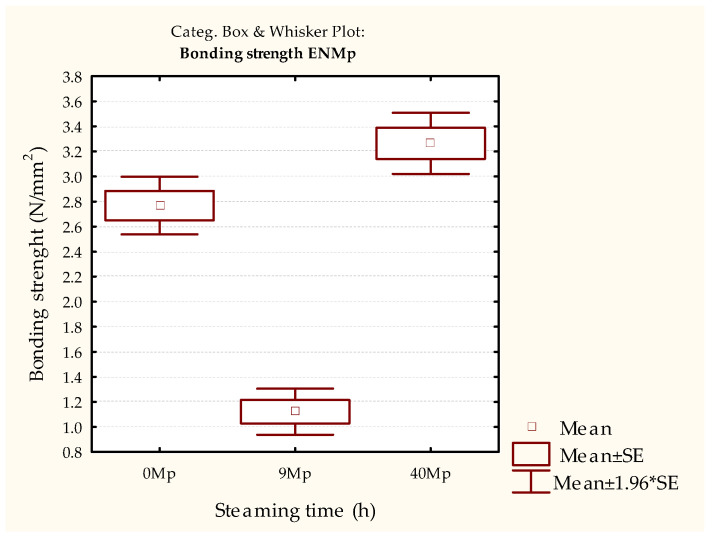
Results of the EN 13354 [30] test of test pieces bonded with PVAc adhesive immersed in water for 24 h.

In contrast, when determining the strength of the bonded joints using the EN 13354 [30] method, the results for differently steamed and non-steamed samples show statistically significant lower strengths obtained in the 9 h steamed samples (Figure 4 and Table 3). When this result is compared with the requirements of the EN 13353 standard [32], it is clear that the joint strength of the 9 h steamed samples does not meet the minimum requirements of 2.5 N/mm^2^.

However, this phenomenon cannot be seen in the samples steamed for 40 h, as their strength increased even in relation to the control set (unmodified samples). This result suggests the positive effect of wood steaming, which, in addition to the match [38], retains the quality of the joint bonded to the PVAc adhesive. However, the initial strength of the bonded joints (Figure 3) did not indicate this possibility.

**Figure 5:**
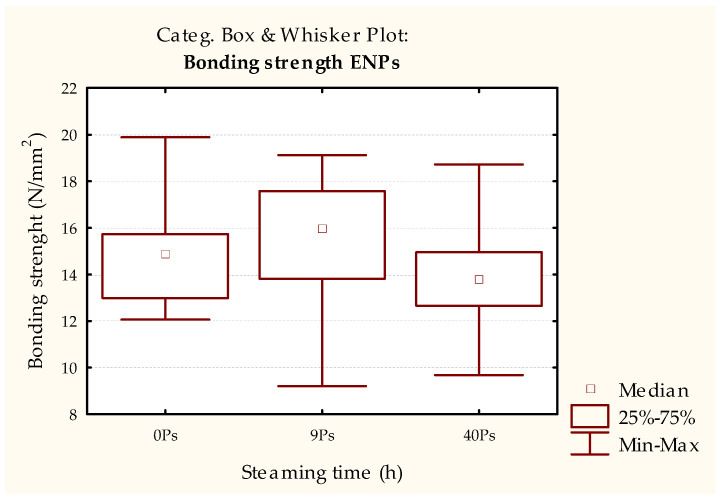
Results of testing conditioned test pieces bonded with PUR adhesive.

**Figure 6:**
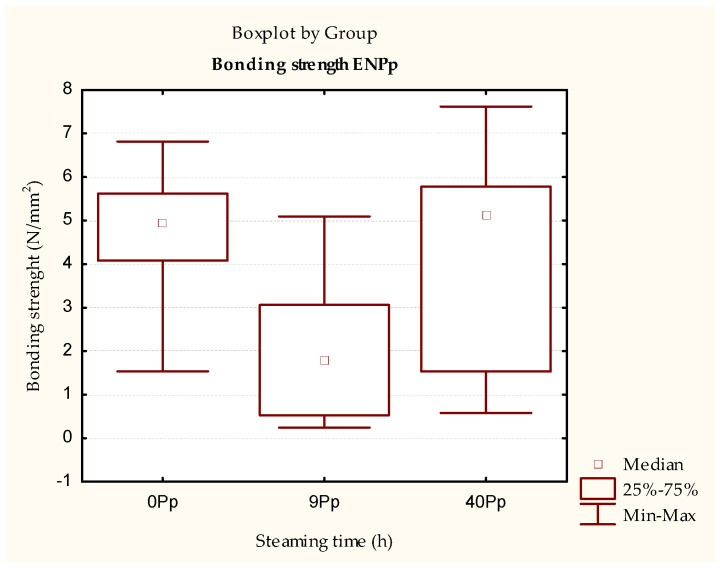
Results of the EN 13354 [30] test of test pieces bonded with PUR adhesive immersed in water for 24 h.

A difference between adhesives can be observed in relation to the initial strength of the PVAc adhesive-bonded samples (Figure 3 and Figure 5). Although there are no statistical differences between the samples of both adhesives, the overall strength of the PUR adhesive, for all types of samples, is ~37% higher on average than the samples bonded with PVAc adhesive (Figure 3, Figure 4, Figure 5 and Figure 6, Table 2, Table 3, Table 4 and Table 5). Additionally, a reverse trend of initial strengths can be observed for the conditioned samples (Figure 3 and Figure 5, Table 2 and Table 4). Samples steamed for 9 h show a slight increase in strength gain, while 40 h of steaming leads to a slight decrease compared to the non-steamed and 9 h steamed samples.

In contrast to this difference, the strength measurement results measured by EN 13354 [30] show the same trend as for PVAc adhesives. The samples steamed for 9 h and bonded with PUR adhesive did not meet the prescribed bond strength of the EN 13353 standard. Their strength was statistically significantly lower than that of the unsteamed samples, and the samples were steamed for 40 h, where the minimum strength requirements were met.

Furthermore, a comparison of the results achieved with the PVAc adhesive shows, as expected, that the PUR adhesive is, on average, more resistant to the influence of water, with mean values ~67% higher for both unmodified and modified samples (Figure 3 and Figure 5, Table 2 and Table 4). 

The reason for the decrease in strength below the prescribed values for both adhesives in all 9 h steamed samples tested according to EN 13354 [30] can be explained by the fact that the steaming of beech wood releases extractives that can affect joint strength [39]. For example, acetic acid, formic acid, and some other acids that are also released in the steaming process [40] are most likely to play an important role in the loss of the bonded joint. In the short-term steaming process, part of the acid stays in the cell lumen, and when retreated with water, the acid from the lumen meets the adhesive in the joint and enhances the destructive action on the bonded joint [41]. After the test, an analysis of the samples confirmed this assumption, where breakage was detected exclusively in the adhesive layer. The acid is mostly alkalized by prolonging the steaming regime, so its influence on the retreated joints is less significant.

### 3.2. Durability of the Bonded Joint

The results of the durability evaluation of a bonded joint should be observed separately from the results of the determination of the strength of a bonded joint because the forms of the test pieces are different. Therefore, the measured amounts are affected by the difference in the construction of the bonded compound, causing the results to be incomparable [42].

**Figure 7:**
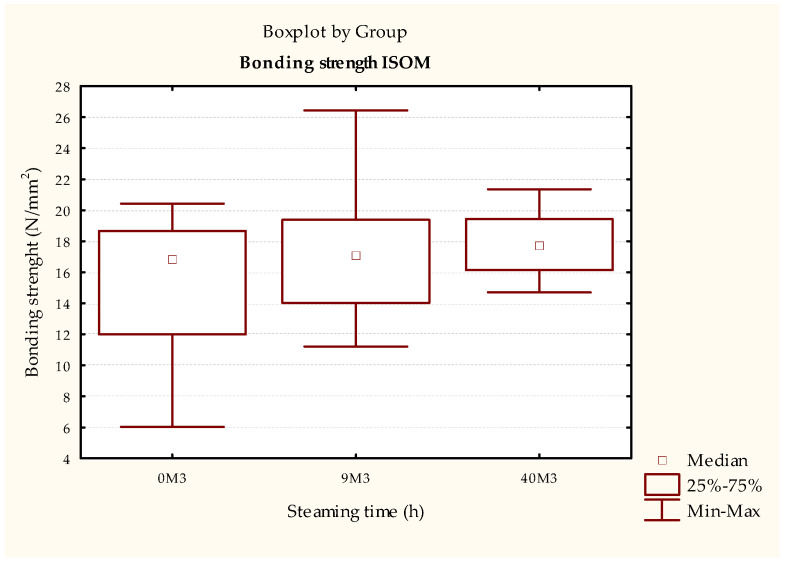
Results of ISO 6238 [31] tests of test pieces bonded with PVAc adhesive aged by ISO 9142 [33]—cycle D6.

**Figure 8:**
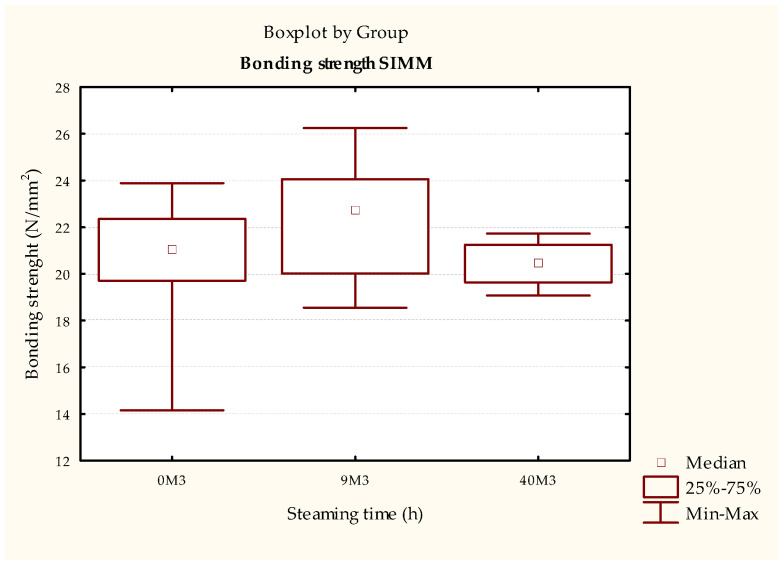
Results of ISO 6238 [31] testing of test pieces bonded with PVAc adhesive aged by humidity change simulation.

The joint durability of the samples bonded with the PVAc adhesive did not show statistically significant differences between the steamed and non-steamed samples tested according to the ISO 9142 [33]—Cycle D6 standard (Figure 7 and Table 6) or the simulation of the environmental conditions method (Figure 8 and Table 7). The strength of the bonded joint was lower when tested according to the ISO 9142 standard [33], which was expected since the PVAc adhesive is not resistant to prolonged exposure to moisture. On the other hand, according to the method of simulation of environmental conditions, the bonded joint is not exposed to water.

When comparing the results of the two methods, 4 h of exposure to water (ISO 9142 [33]—cycle D6) already leads to a significant reduction (Figure 7 and Figure 8, Table 6 and Table 7) in the total strength of all test samples. However, the most significant differences occur in the control test pieces; then somewhat smaller differences occur in the 9 h steamed test pieces, and the smallest in the 40 h steamed test pieces.

Although there is no statistically significant difference, there is a noticeable trend of decreased scattering of results (Figure 7), i.e., increasing the uniformity of strength, which is a positive result.

**Figure 9:**
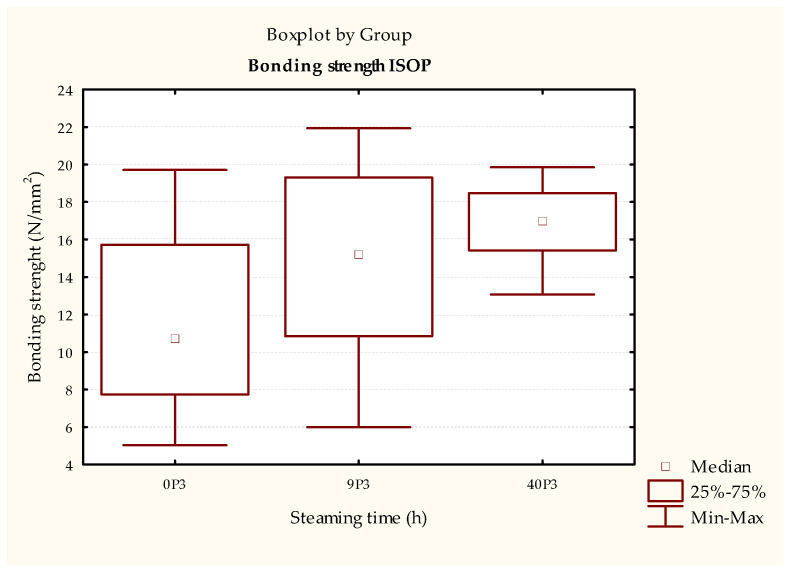
Results of the ISO 9142 [33] testing of test pieces bonded with PVAc adhesive aged by ISO 9142 [33]—cycle D6.

**Figure 10:**
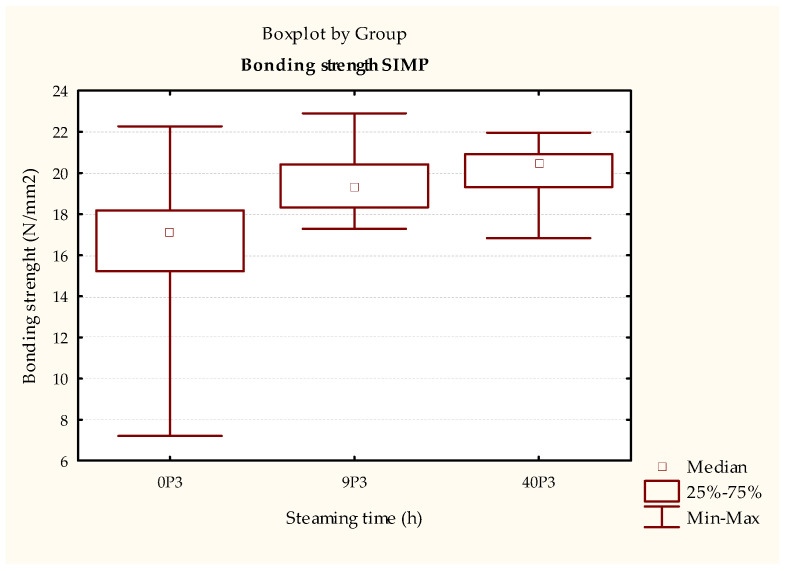
Results of ISO 9142 [33] tests of test pieces bonded with PUR adhesive aged by humidity change simulation.

The test results of the durability of the bonded joint using PU adhesive in the samples treated according to ISO 9142 [33] cycle D6 (Figure 9 and Table 8) show a statistically significant increase in strength of the samples steamed for 40 h compared to non-steamed samples. Using the simulation of realistically possible changes in the humidity method (Figure 10 and Table 9), the same trend is observed, that is, the strength of the bonded compound is higher in the steamed samples (both 9 and 40 h). An analysis of these results shows that increasing the steaming time increases the durability of the bonded joint in samples bonded with PU adhesive. This result is supported by the literature, where Unsal and Ayrilmis concluded that shrinkage and swelling in steamed wood are less pronounced than in non-steamed wood due to lower EMC. Thus, the steaming process can lead to better results in the durability of bonded joints.

From the above-presented results and discussion, it is quite easy to conclude that due to the effect of wood steaming, visible changes have certainly occurred, and a constant amount of dry strength is secured regardless of the duration of steaming, which is an excellent result of this modification, although its primary aim is to even out the color of the wood. Another visible contribution is expressed only in contact with PUR adhesive. In addition to the primary result, we obtained a significant increase in the durability of the glued joint, confirmed by two methods. If we try to consider/point out mechanisms that led to such good results, we will have to look for them in the achieved adhesion mechanisms and in the “matching” of the properties of wood and PUR adhesive. With the achieved amount of results, it is difficult to reliably determine in which of the 5 adhesion mechanisms of PUR and wood there was an increase in adhesion results. Are these changes in the surface morphology, i.e., the release of surface openings in the wood, that would result in an increased mechanical share of adhesive mechanisms, or are we talking about changes in the chemical structure that increase the diffusion of adhesive polymer chains into the wood substance and the realization of a greater share of chemical bonds? We should certainly not ignore the possibility of increasing the energy of the wood surface, which would then be capable of increasing adhesion to PUR. Perhaps it is simply a matter of increasing the polarity of the wood, which then contributes to the overall adhesion with its electrostatic changes.

Some mechanisms could still be ruled out if, for example, we take into account the extremely high polarity of the water molecules participating in the durability tests, but the other mechanisms should be additionally tested in order to more reliably determine which adhesion mechanisms contributed to such a good result in bond durability. 

Although it has been proven that improvements do exist with the use of PUR adhesive, testing with PVAc shows that these improvements will not always be beneficial if the quality of the adhesive is not good enough.

**Figure 11:**
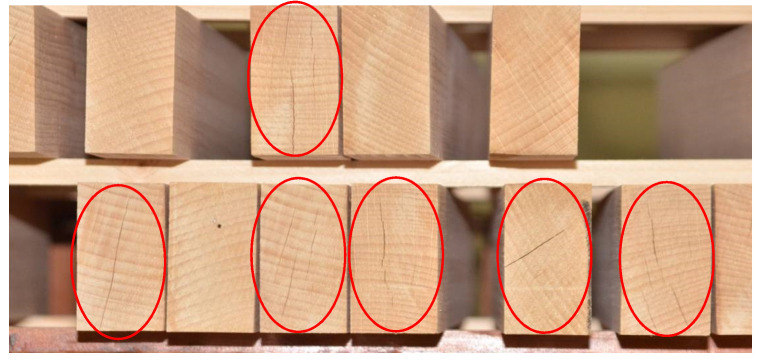
Cracks occur in reconditioned control samples exposed to high relative humidity for seven days.

**Figure 12:**
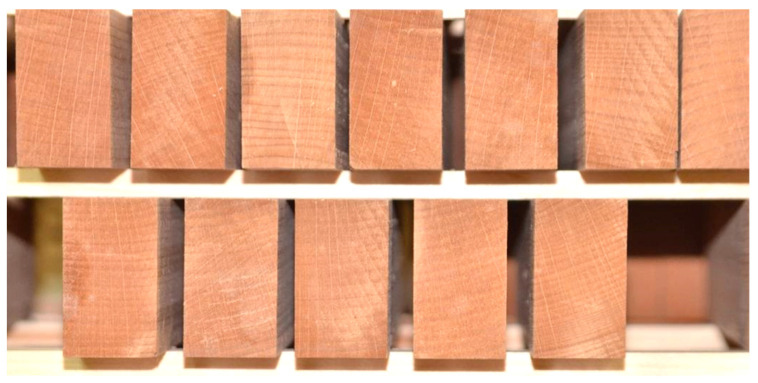
No cracks on reconditioned 9 h steamed samples exposed to high relative air humidity for seven days.

**Figure 13:**
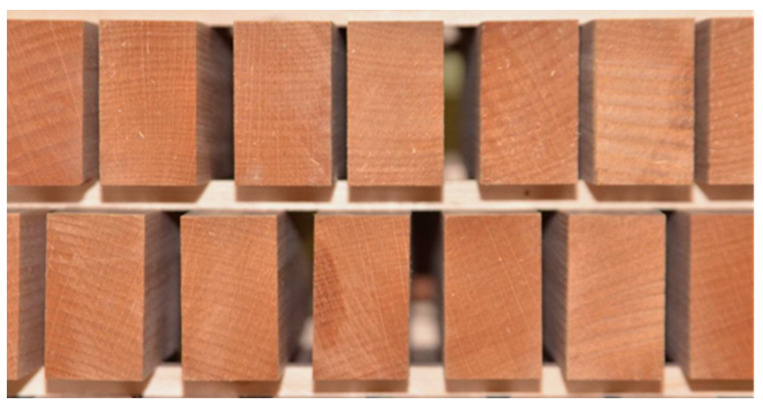
No cracks on reconditioned 40 h steamed samples exposed to high relative air humidity for seven days.

The last result was interesting in terms of determining the dimensional changes of the modified wood, which could be a very positive result even at the initial exposure of the samples to increased air humidity. After the simulation of an indoor flood in which samples were exposed to 85% relative air humidity for seven days, the result obtained provides additional support for our conclusions and findings of Unsal and Ayrilmis [42] as well as a guide for further investigation of the dimensional changes of such modified wood. From the figures below, it is clear that cracks are present in the unmodified wood samples, while in the modified samples, this could not be noticed (Figure 11, Figure 12 and Figure 13). This was previously noted, but only for the drying process that follows steaming [4].

## 4. Conclusions

The test results of the achieved strengths indicate that the steaming modification does not adversely affect the bonding process or the bonded joint.

Submersion in water leads to a decrease in bond strength below the prescribed values in 9-h steamed wood samples bonded with PVAc and PUR adhesive. Extending the time of exposure of wood to steam causes the return of the bond strength level close to the initial size of the controlled, non-steamed samples.

Increasing the steaming time of the wood does not increase the durability of the PVAc-bonded joints.

Increasing the steaming time of the wood significantly increases the durability of the PU-bonded joint.

Observing the overall test results, it can be concluded that the steaming of the beech wood changes the structure of the wood matter, which contributes to maintaining the strength of the bonded joint under enhanced aging processes.

## Figures and Tables

**Table 1:** The method of marking test pieces.

TYPE OF EXAMINATION	MARK
**1. Test of bonding strength**	
*Bonding quality—Test method EN 13354* [30]	**EN**
**2. Durability of the bonded joint**	
*(a) Testing of durability by standard*	**ISO**
*(b) Testing the durability by simulating conditions*	**SIM**
**3. Steaming time**	
*(a) Not steamed samples*	**0**
*(b) Samples steamed for* 9 h	**9**
*(f) Samples steamed for* 40 h	**40**
**4. Type of adhesive**	
*(a) PVAc adhesive*	**M**
*(b) PUR adhesive*	**P**
**5. Other terminology**	
*(a) Mass*	**m**
*(b) Volume*	**v**
*(c) Conditioned samples* (50 ± 5% r.h., 23 ± 2 °C)	**s**
*(d) Samples of the dry condition*	**S**
*(e) Impregnated samples and immersed in water for* 24 h	**V**
*(f) Samples exposed to a relative humidity of* 70% *(NaCl)*	**7**
*(g) Samples exposed to a relative humidity of* 30% *(MgCl*_2_ *+* 6*H*_2_*O)*	**3**
*(h) Samples exposed to relative humidity* 85 ± 3%	**8**
*(i) Samples immersed in water for* 24 h	**p**

**Table 2:** Kruskal–Wallis test of conditioned test pieces bonded with PVAc adhesive.

Dependent: ENMs	Multiple Comparisons *p*-Values (2-Tailed); ENMs (Bonding Strength (N/mm^2^)) Independent (Grouping) Variable: Designation Kruskal-Wallis Test: H (2, N = 60) = 1.206397 *p* = 0.5471		
	0 Ms R: 28.650	9 Ms R: 28.850	40 Ms R: 34.000
0 Ms		1.000000	0.998036
9 Ms	1.000000		1.000000
40 Ms	0.998036	1.000000	

**Table 3:** Scheffe test of test pieces bonded with PVAc adhesive immersed in water for 24 h.

Designation	Scheffe Test; Variable: ENMp (Bonding Strength (N/mm^2^)) The Marked Differences Are Significant at *p* < 0.0500		
	(1) M = 2.7673	(2) M = 1.1218	(3) M = 3.2644
0 Mp (1)		0.000000	0.010002
9 Mp (2)	0.000000		0.000000
40 Mp (3)	0.010002	0.000000	

**Table 4:** Statistical test of conditioned test pieces bonded with PUR adhesive.

Variable	Analysis of Variance (Bonding Strength (N/mm^2^)) Marked Effect Are Significant at *p* < 0.0500							
	SS Effect	df Effect	MS Effect	SS Error	df Error	MS Error	F	*p*
ENPs	18.94624	2	9.473118	312.1804	56	5.574651	1.699320	0.192098

**Table 5:** Statistical test of test pieces bonded with PUR adhesive immersed in water for 24 h.

Dependent: ENPp	Multiple Comparisons *p*-Values (2-Tailed); ENPp (Bonding Strength (N/mm^2^)) Independent (Grouping) Variable: Designation Kruskal-Wallis Test: H (2, N = 81) = 23.58860 *p* = 0.000		
	0 Pp R: 50.464	9 Pp R: 22.040	40 Pp R: 48.464
0 Pp		0.000034 *	1.000000
9 Pp	0.000034 *		0.000134 *
40 Pp	1.000000	0.000134 *	

* The bold numbers represent statistical results showing a statistically significant difference between the tested samples.

**Table 6:** Statistical test of test pieces bonded with PVAc adhesive aged by ISO 9142 [33]—cycle D6.

Dependent: ISOM	Multiple Comparisons *p*-Values (2-Tailed); ISOM (Bonding Strength (N/mm^2^)) Independent (Grouping) Variable: Designation Kruskal-Wallis Test: H (2, N = 73) = 3.550398 *p* = 0.1695		
	0M3 R: 30.960	9M3 R: 38.296	40M3 R: 42.524
0M3		0.638539	0.196751
9M3	0.638539		1.000000
40M3	0.196751	1.000000	

**Table 7:** Statistical test of test pieces bonded with PVAc adhesive aged by humidity change simulation.

Dependent: SIMM	Multiple Comparisons *p* Values (2-Tailed); SIMM (Bonding Strength (N/mm^2^)) Independent (Grouping) Variable: Designation Kruskal–Wallis Test: H (2, N = 73) = 8.917836 *p* = 0.0116		
	0M3 R: 34.333	9M3 R: 46.680	40M3 R: 28.000
0M3		0.094932	0.950187
9M3	0.094932		0.013192 *
40M3	0.950187	0.013192 *	

* The bold numbers represent statistical results showing a statistically significant difference between the tested samples.

**Table 8:** Statistical test of test pieces bonded with PVAc adhesive aged by ISO 9142 [33]—cycle D6.

Dependent: ISOP	Multiple Comparisons *p*-Values (2-Tailed); ISOP (Bonding Strength (N/mm^2^)) Independent (Grouping) Variable: Designation Kruskal-Wallis Test: H (2, N = 86) = 15.24246 *p* = 0.0005		
	0P3 R: 30.033	9P3 R: 46.500	40P3 R: 55.577
0P3		0.031941 *	0.000404 *
9P3	0.031941 *		0.524660
40P3	0.000404 *	0.524660	

* The bold numbers represent statistical results showing a statistically significant difference between the tested samples.

**Table 9:** Statistical test of test pieces bonded with PUR adhesive aged by humidity change simulation.

Dependent: SIMP	Multiple Comparisons *p*-Values (2-Tailed); SIMP (Bonding Strength (N/mm^2^)) Independent (Grouping) Variable: Designation Kruskal–Wallis Test: H (2, N = 82) = 34.22592 *p* = 0.0000		
	0P3 R: 21.867	9P3 R: 48.077	40P3 R: 57.577
0P3		0.000120 *	0.000000 *
9P3	0.000120 *		0.451074
40P3	0.000000 *	0.451074	

* The bold numbers represent statistical results showing a statistically significant difference between the tested samples.

## Data Availability

The data presented in this study are available upon request from the corresponding authors.
